# methylCC: technology-independent estimation of cell type composition using differentially methylated regions

**DOI:** 10.1186/s13059-019-1827-8

**Published:** 2019-11-29

**Authors:** Stephanie C. Hicks, Rafael A. Irizarry

**Affiliations:** 1 Department of Biostatistics, Johns Hopkins Bloomberg School of Public Health, 615 N Wolfe St,, Baltimore, USA; 2 Department Data Sciences, Dana-Farber Cancer Institute, 450 Brookline Ave,, Boston, USA; 3 Department of Biostatistics, Harvard T.H. Chan School of Public Health, 677 Huntington Ave, Boston, USA

**Keywords:** DNA methylation, Whole blood, Cell composition, Microarray, HumanMethylation27 BeadChip, HumanMethylation450 BeadChip, Whole genome bisulfite-sequencing, Reduced representation bisulfite-sequencing

## Abstract

A major challenge in the analysis of DNA methylation (DNAm) data is variability introduced from intra-sample cellular heterogeneity, such as whole blood which is a convolution of DNAm profiles across a unique cell type. When this source of variability is confounded with an outcome of interest, if unaccounted for, false positives ensue. Current methods to estimate the cell type proportions in whole blood DNAm samples are only appropriate for one technology and lead to technology-specific biases if applied to data generated from other technologies. Here, we propose the technology-independent alternative: *methylCC*, which is available at https://github.com/stephaniehicks/methylCC.

## Background

DNA methylation (DNAm) is a type of chemical modification occurring at CpG dinucleotide sites that is involved in controlling gene expression and has been shown to play an important role in distinguishing cell lineages [1]. High-throughput DNAm assays have been widely applied among researchers as well as large consortia to further our understanding of basic biology and health implications [2]. However, a major challenge in extracting information from these DNAm datasets is variability introduced from intra-sample cellular heterogeneity observed in samples of heterogeneous cell composition. Specifically, individual cell types encode unique cell type-specific DNAm signatures to distinguish between the cell lineages. Therefore, when we measure DNAm on samples with a heterogeneous cell composition, we actually observe a convolution of the DNAm profiles of each cell type [3]. It is common for variability in cell type proportions to explain most of the observed sample-to-sample variability.

Cell composition induced variability is particularly problematic in epigenome-wide association studies (EWAS) [4] because, due to convenience, these are most frequently performed on whole blood, a highly heterogeneous tissue. In a seminal paper, Houseman et al. [3] describe a statistical method that accurately estimates the relative proportions of cell type components in whole blood. Jaffe et al. [5] used this approach to demonstrate that reported age-related changes of blood DNAm profiles [6–12] could be explained with high levels of confounding between age-related variability and cell composition, demonstrating the importance of accounting for this source of variability. As the consequential effect of this source of variability started to be recognized, interest in statistical methods for estimating and accounting for intra-sample cellular heterogeneity grew accordingly. There are currently two major types of approaches. The first, originally developed by Houseman et al. [3], assumes that the observed heterogeneous blood profiles are a linear combination of the cell type-specific DNAm profiles, assumes these DNAm profiles are known, and then estimates the unknown proportions using a standard estimation procedures. To be able to assume cell type-specific DNAm profiles are known, a rather complex experiment, in which cells of the same cell type are sorted and then used to obtain high-throughput measurements of the reference samples, is conducted. Methods that make use of the sorted cell type-specific DNAm profiles are referred to as *reference-based*. Alternatively, other methods that do not use external reference profiles, referred to as *reference-free* methods, have been developed for DNAm data [13, 14] and for more general types of data such as Surrogate Variable Analysis (SVA) [15], or Remove Unwanted Variability (RUV) [16] to account for batch effects [17].

Reference-based approaches have been shown to greatly outperform reference-free procedures [18]. Here, we consider them to be the state of the art. However, in this paper, we demonstrate that a limitation of reference-based approaches is the presence of a technology-specific bias, which can influence the estimates of cell composition; namely, when using cell type-specific DNAm profiles measured using one technology, for example a microarray platform, to estimate the cell type proportions in samples measured from another technology, for example a sequencing platform. Here, we introduce a statistical method, referred to as *methylCC*, that removes this technical bias using a latent variable model and accurately estimates the cell composition in a platform-agnostic manner. To achieve this, we identify regions of the genome in which each cell type are either clearly methylated or unmethylated, and we model these as latent states. These latent states are biologically driven and therefore technology-independent, which allows us to estimate binary, platform-independent profiles that can be successfully be applied across technologies. To study the improvements in estimates of cell composition using methylCC, we evaluated the difference between the true and estimated proportions of cell types with a Monte Carlo simulation. Specifically, we studied how using cell type-specific DNAm profiles measured on a microarray platform to estimate the cell type proportions in samples measured on a sequencing platform can lead to inaccurate estimates of cell composition. Furthermore, we demonstrate how our platform-agnostic approach provides an overall improvement in estimates of cell composition. Although due to the availability of data all our examples are from whole blood, the approach can be generalized to other tissues.

## Results

Consider a set of high-throughput data *Y*_*ij*_ representing a heterogeneous tissue sample, such as whole blood, from *i*∈(1,…,*N*) individuals containing DNAm measurements at CpG sites *j*∈(1,…,*J*). Suppose the heterogeneous tissue is a combination of *K* cell types, which we index with *k*∈(1…*K*). Houseman et al. [3] proposed the following statistical model to estimate the proportions of *K* cell types in whole blood DNAm samples, for each individual *i*:

1 $$  \boldsymbol{Y}_{i} = \sum_{k=1}^{K} \pi_{ik} \boldsymbol{X}_{k} + \boldsymbol{\varepsilon_{i}}.  $$

Here *π*_*ik*_ represents the proportion of cell type *k* in individual *i*, which is the parameter of interest. The ***X***_*k*_ represents the *k*th cell type-specific DNAm profile with measurements at the same *J* CpG sites as ***Y***_*i*_. The measurement error and other unexplained biological variability is represented by ***ε***_*i*_. The cell type proportions for individual *i* are assumed to be nonnegative, *π*_*ik*_≥0, and sum to 1, $\sum _{k=1}^{K} \pi _{ik} = 1$. To develop a practical tool, Houseman et al. [3] sorted whole blood samples into *K*=6 cell types that make up the majority of this tissue and obtained a DNAm profile for each cell type. They used Illumina’s HumanMethylation27 BeadChip (Illumina 27K), which measures DNAm at approximately 27,000 CpG sites [19]. This experimental data provided plug-in estimates for the cell type-specific DNAm profile, ***X***_*k*_, and with these in place then they estimated the *π*_*ik*_ using a constrained least square algorithm. Soon after the development of this method, Illumina released a new platform that measured approximately 450,000 CpG sites: the HumanMethylation450 BeadChip (Illumina 450K) [20]. Jaffe et al. [5] leveraged publicly available data of sorted cell types measured with this new Illumina 450K platform [1] to implement the Houseman et al. method [3].

Although the Illumina 450K microarray platform has been the most widely used platform, two new sequencing technologies are being increasingly adapted by the research community: Whole-genome Bisulfite Sequencing (WGBS) and Reduced Representation Bisulfite Sequencing (RRBS) [21]. Furthermore, Illumina has recently released a new version of their BeadChip, which measures approximately 850,000 CpG sites. However, similar experiments with sorted cells processed at the same time are not yet available from these new platforms, which implies we do not have plug-in estimates for ***X***_*k*_ on these platform technologies. Currently, the only way the Houseman et al. approach [3] can be applied to DNAm data measured on these new platforms is by assuming that the cell type-specific DNAm profiles ***X***_*k*_ derived for the 450K platform applies to others. Here, we show this assumption does not hold.

### Across platforms estimates are inaccurate

**Fig. 1:**
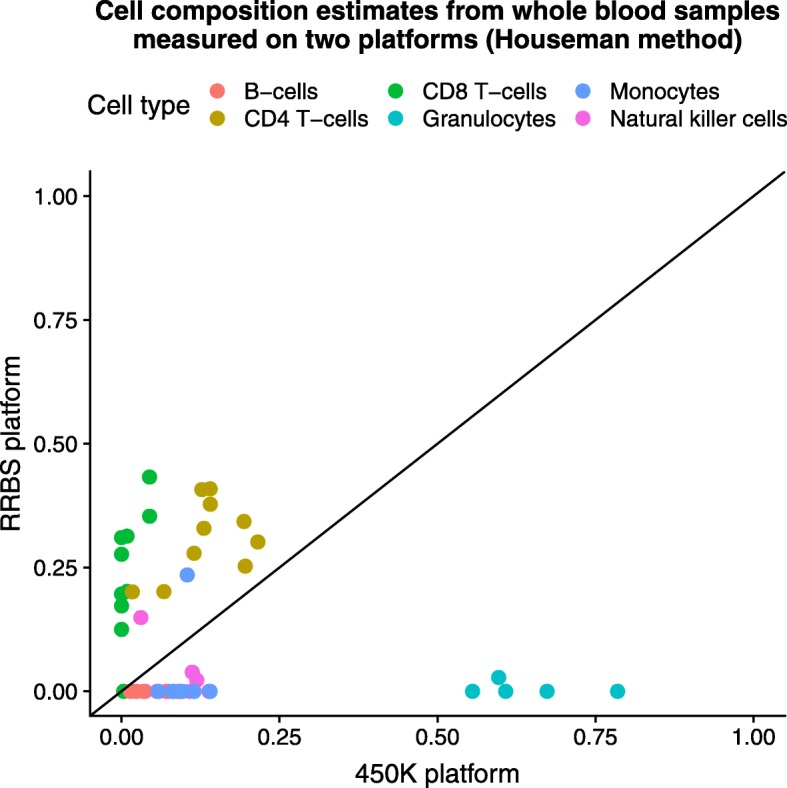
Across platforms estimates are inaccurate. Cell composition estimates (*K*=6 cell types) from *N*= 10 whole blood samples (*two-platform dataset* – GEO Accession GSE95163) measured on the Illumina 450k microarray platform (*x*-axis) and the RRBS platform*(y-*axis). The statistical method proposed by Houseman et al. [3], as implemented by Jaffe et al. [5], and was used to estimate the cell composition

To determine if the Houseman et al. method [3], as implemented by Jaffe et al. [5], which was specifically developed for the Illumina 450K array platform, is applicable across platforms, we obtained a dataset for which whole blood samples from *N*=10 adult males were run on both the Illumina 450K and RRBS platforms (referred to below as the *two-platform dataset*). We applied the Houseman method to the whole blood samples in the *two-platform dataset* and expected similar cell composition estimates for each individual across platforms as these were the same whole blood samples just measured on two platforms. Because the Houseman method has been shown to provide reliable cell composition estimates for DNAm data measured on the Illumina 450K platform, in this specific case we considered the cell composition estimates from the Houseman method to be the gold-standard or ground truth, as done by Rahmani et al. [22]. However, we found that the resulting cell composition estimates between the *N*=10 whole blood samples measured on the Illumina 450K and RRBS platforms did not agree (Fig. 1).

To determine the cause of this disagreement, we examined this dataset more closely and found two limitations with the Houseman approach when applied to technologies other than the Illumina microarrays: (1) DNAm measurements vary across platforms and (2) different platforms measure different CpGs. These two limitations are discussed in the following two sections, respectively. We then describe a statistical solution to overcome these two limitations using a general latent class model to estimate the cell composition of heterogeneous samples agnostic to platform technology. We also provide a software implementation of our method available at https://bioconductor.org/packages/release/methylCC.

### DNAm measurements vary across platforms

**Fig. 2:**
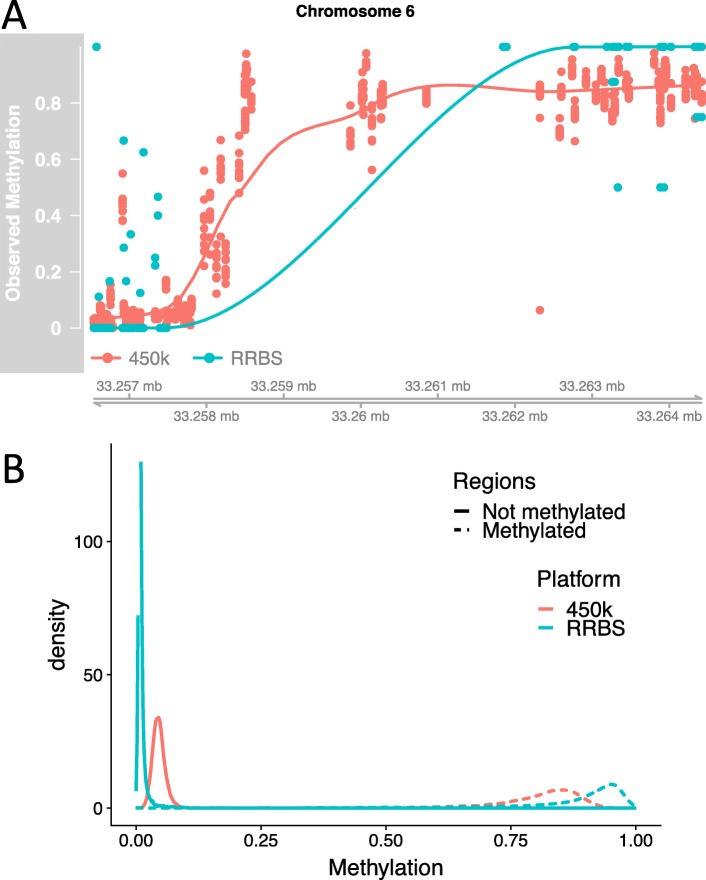
Evidence for platform-dependent bias. **a** Observed DNAm levels from one region in same *N*=10 individuals (*two-platform dataset* – GEO Accession GSE95163) measured on two platform technologies: Illumina 450k (red) and RRBS (blue). **b** Density of DNAm levels measured on the 450k platform (red) and RRBS platform (blue) in regions that are either methylated (dashed line) or not methylated (solid line). Regions were identified by searching for regions in purified whole blood cell types from Reinius et al. [1] that appeared either methylated or not methylated in all six purified cell types

The first limitation is that there is a platform-dependent bias. We can observe this bias by simply plotting and comparing the raw DNAm measurements using the whole blood samples in the *two-platform dataset*. We commonly observe genomic regions in which both platforms seem to indicate a change from unmethylated to methylated states, but the observed DNAm levels differ substantially across platforms (for example, Fig. 2a). A more systematic demonstration is obtained by first using the reference cell sorted dataset [1] to identify regions that are clearly unmethylated in all purified cell types and regions that are clearly methylated in all purified cell types, and then plotting the empirical DNAm distribution across all whole blood samples within these regions for both platforms (Fig. 2b) and noting the different distributions. We note in particular that observed DNAm levels measured on RRBS tends to have values closer to 0 and 1, compared to the Illumina 450K array attenuating these values away from the edges.

### Different platforms measure different CpGs

**Fig. 3:**
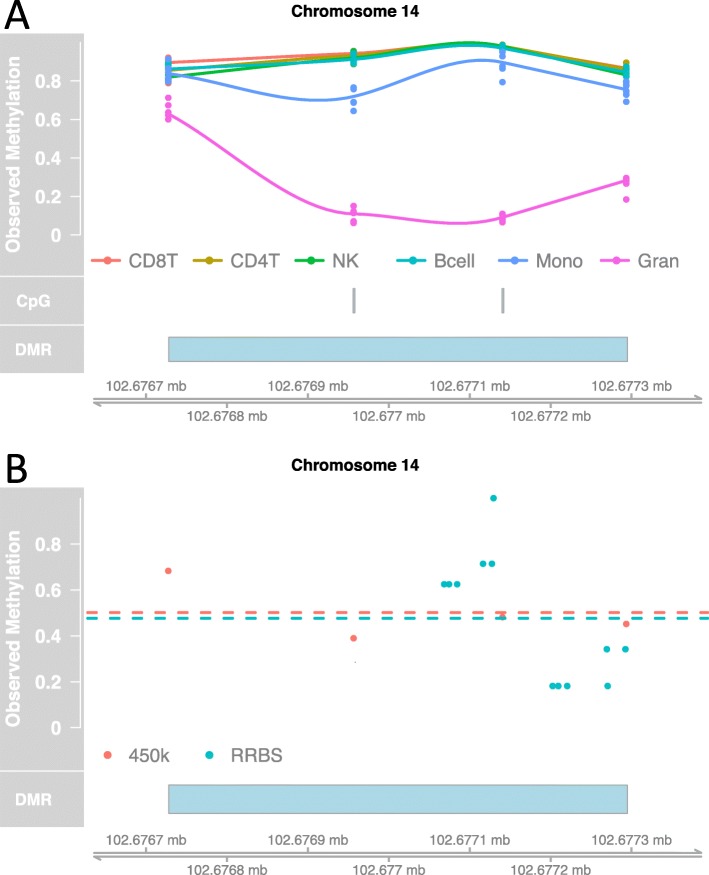
Example of Houseman cell type-specific CpGs measured on the 450k microarray platform, but not measured on the RRBS platform. **a** The cell type-specific CpG and the cell type-specific region of CpGs (or differentially methylated regions, DMR) were both identified using the purified CD8T cell, CD4T cell, Natural Killer (NK), B cell, Monocytes (Mono) and Granulocytes (Gran) cell types from [1]. **b** In the whole blood DNAm data from one individual (GEO Accession GSE95163) measured on two platforms (450k and RRBS), the cell type-specific CpG from Houseman et al. [3] is not measured on the RRBS platform. However, using a cell type-specific region (DMR), we are able to measure the methylation level by averaging across the region in both the 450k and RRBS platform for this sample (dotted lines)

The second limitation is that different platforms measure different CpGs. The human genome contains over 20,000,000 CpG sites, and each platform includes a subset of these which, for logistical reasons, differs across platforms. For example, RRBS [21] uses restriction enzymes to enrich for the areas of the genome that have a high CpG content, while the Illumina 450k platform selects CpG sites that are more uniformly distributed across the genome. Therefore, to apply the Houseman model to samples measured on platforms other than the Illumina 450K array, we have to restrict ourselves to the intersection of the CpGs measured Illumina 450K array and the alternative platform, because the *j*^*th*^ CpG in the whole blood sample ***Y***_*i*_ must match the *j*^*th*^ CpG in the cell type-specific DNAm profile ***X***_*k*_. As a result, in our 10 RRBS samples we only have measurements from 102 of the 600 CpGs in the cell type-specific DNAm profiles used by the 450K implementation of the Houseman method. This results in a loss of power since informative cell type-specific CpGs may be left out (for example Fig. 3).

### methylCC estimates cell composition in DNAm samples agnostic to platform technology

To adjust for the platform-specific biases, we introduce a model that accounts for these biases directly and models methylation states using latent variables. To account for the fact that different platforms measure different CpG sites, we model the latent classes at the region level rather than the CpG level. Specifically, we propose the following statistical model:

2 $$  \boldsymbol{Y}_{i} = \sum_{k=1}^{K} \pi_{ik} \left\{ (1-\boldsymbol{Z}_{k}) \boldsymbol{\delta}_{0} + \boldsymbol{Z}_{k} \boldsymbol{\delta}_{1} \right\} + \boldsymbol{\varepsilon}_{i}  $$

where ***Y***_*i*_ is the observed DNAm level in the heterogeneous tissue, in this case whole blood, for the *i*^*th*^ individual *i*∈(1,…,*N*), but now measured DNAm levels in *R* genomic regions *r*∈(1,…,*R*), as opposed to *J* individual CpGs in the Houseman model. Similar to the Houseman model, *π*_*ik*_ represents the proportion of cell type *k* in individual *i*, which is the parameter of interest. In addition, we assume that the cell type proportions for individual *i* are nonnegative, *π*_*ik*_≥0, and sum to 1, $\sum _{k=1}^{K} \pi _{ik} = 1$. Here, ***Z***_*k*_=(*Z*_1*k*_,…,*Z*_*Rk*_) is a vector of latent variables for the *k*^*th*^ cell type where each latent variable, *Z*_*rk*_, is an indicator that is equal to 1 if the region *r* is methylated in cell type *k* and 0 otherwise. The platform-specific biases are represented with random effects ***δ***_0_=(*δ*_0,1_,…,*δ*_0,*R*_) and ***δ***_1_=(*δ*_1,1_,…,*δ*_1,*R*_), which are assumed to follow multivariate normal distributions $N\left (\alpha _{0} \boldsymbol {1}, \sigma _{0}^{2} I_{(R\times R)}\right)$ and $N\left (\alpha _{1} \boldsymbol {1}, \sigma _{1}^{2} I_{(R\times R)}\right)$, respectively. Measurement error and other unexplained biological variability is represented with ***ε***_*i*_, which we assume follows a multivariate normal distribution *N*(0,*τ*^2^*I*_(*R*×*R*)_). Note that in our model the random effects ***δ***_0_ and ***δ***_1_ are assumed to be platform-dependent: they represent the technology-dependent bias with different mean and variances in different platforms (Fig. 2b). However, the ***Z***_*k*_s are not platform-dependent: they are latent classes determined by biology.

The statistical model in Eq. 2 can be thought of as a generalization of Eq. 1 if we restrict the Houseman approach to only include CpGs that are either methylated or unmethylated in each cell type. In this case, region *r* would simply be a single CpG site and the *k*^*th*^ cell type-specific DNAm profile, ***X***_*k*_, would be defined by *X*_*rk*_=*δ*_0,*r*_ if region *r* is unmethylated and *X*_*rk*_=*δ*_1,*r*_ if region *r* is methylated.

A significant advantage of our model is that instead of directly measuring the cell type-specific DNAm profiles, ***X***_*k*_, for each platform, we account for region-to-region variability using a latent random variable and therefore do not need to measure it directly with each new platform. Instead, all we need is to identify *R* regions for which each *k*^*th*^ cell type is either clearly methylated (*Z*_*rk*_=1), or not methylated (*Z*_*rk*_=0) for *r*∈(1,…,*R*). We define ***Z*** to be the matrix with entries *Z*_*rk*_ in the *r*th row and *k*th column, which needs to be full rank for the parameters of interest, *π*_*ik*_ to be identifiable. Because ***Z*** is entirely determined by biology, not by the platform technology, we only have to identify these regions once for each type of heterogeneous (biological) sample. This requires experimental data from cell sorted samples measured on only one platform. To demonstrate the utility this approach for estimating cell composition in whole blood samples, we searched for these genomic regions in the purified cell type data described in [1], which were measured on the Illumina 450K array platform. This dataset includes B cells, monocytes, granulocytes, CD8T cells, CD4T cells and natural killer (NK) cells. We identified *R*=210 regions satisfying our criteria (Additional file 1: Figure S1). Finally, with the *R* regions in place, the estimation of the proportion of cell types, *π*_*ik*_, reduces to a missing data problem. We use an EM algorithm with a constrained linear model to estimate the parameters $\boldsymbol {\theta } = \left (\alpha _{0}, \alpha _{1}, \sigma _{0}^{2}, \sigma _{1}^{2}, \tau ^{2}\right)$ and ***π***_*i*_=(*π*_*i*1_,…,*π*_*iK*_) for individuals *i*∈(1,…,*N*) (see the "Methods" section for complete details on estimation procedure).

### methylCC improves estimates of cell composition of DNAm samples measured on other platform technologies

**Fig. 4:**
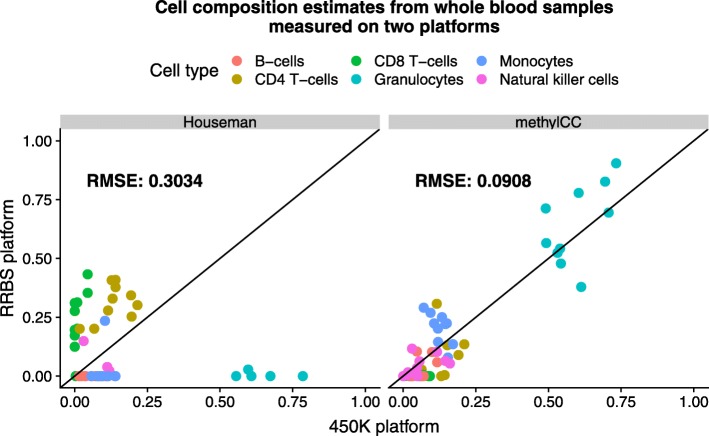
methylCC: A latent variable model with region-specific and platform-dependent random effects improves estimates of cell composition. Cell composition estimates from *N*= 10 whole blood samples (*K* = 6 cell types) measured on the Illumina 450k microarray platform (*x*-axis) and the RRBS platform (*y*-axis). Two methods were used to estimate the cell composition: (1) the model proposed by Houseman et al. [3], as implemented by Jaffe et al. [5], that was developed for samples measured on the Illumina 450k microarray platform (left), and (2) our proposed method that is independent of platform technology (right). Root mean squared error (RMSE) is the difference between the cell composition estimates for whole blood DNAm samples measured on the Illumina 450k array platform and on the RRBS platform (averaged across cell types). Within each plot, the RMSE is shown for the reference-based method from Houseman et al. [3] (left) and our proposed model (methylCC) (right)

To demonstrate the improvements in the estimates of cell composition provided by our platform-agnostic approach, we applied our method to the *two-platform dataset*. Specifically, we fit our model to the 10 whole blood samples measured on both the Illumina 450K array and RRBS platforms. Similar to Fig. 1, we considered the estimates of cell composition from the Houseman model in the Illumina 450K samples to be the gold-standard reference. In Fig. 1, we demonstrated that directly applying the Houseman approach [3], as implemented by Jaffe et al. [5], to the RRBS data led to biased cell composition estimates. However, our new approach substantially improves estimates of cell composition (Fig. 4).

Furthermore, we evaluate the performance of our platform-agnostic approach with the goal of estimating the proportion of cell types in heterogeneous tissue samples. Here, we performed a Monte Carlo simulation study to illustrate the improvements in estimates of cell composition by our platform-agnostic approach compared to the Houseman approach for heterogeneous samples measured on a sequencing platform (described in detail in the Methods Section). For the simulations study, we created cell type-specific DNAm profiles for a microarray platform, $\boldsymbol {X}_{k}^{450K}$, and a sequencing platform, $\boldsymbol {X}_{k}^{RRBS}$, by simulating platform-dependent random effects with different means and variances (Additional file 11: Figure S2A). Then, we simulate whole blood samples with a relative proportion of cell types ***π***_*i*_ and measurement error ***ε***_*i*_ to create the observed DNAm level in whole blood samples measured on in the 450k array platform $\boldsymbol {Y}_{i}^{450k}$ and the RRBS platform $\boldsymbol {Y}_{i}^{RRBS}$. We estimate the cell composition in the whole blood samples measured on both platform using the reference-based Houseman method and our platform-agnostic method. Then, we evaluate the difference between the true and estimated proportion of cell types using either our approach or the Houseman approach.

For whole blood samples measured on the 450K array platform, we found the Houseman approach, which was specifically developed for the array platform, and our approach perform similarly (Additional file 1: Figure S2B). However, for whole blood samples measured on a sequencing platform, our platform-agnostic model results in significantly improved estimates of cell composition (Additional file 1: Figure S2C). This is because our model accounts for the platform-specific biases directly and models methylation states using latent variables.

### methylCC accurately estimates cell composition of DNAm samples measured on WGBS platforms

**Fig. 5:**
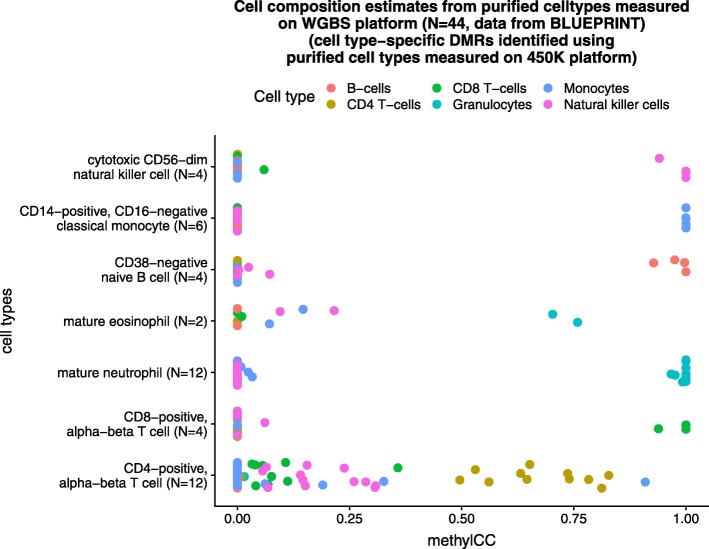
methylCC accurately estimates cell composition of DNAm samples measured on WGBS platforms. Cell composition estimates using *N*=44 WGBS samples from the BLUEPRINT Epigenome Database [23] from seven purified whole blood cell types, specifically B cells, CD4T cells, CD8T cells, neutrophils, eosinophils, monocytes, and natural killer cells. For a given WGBS sample (e.g., CD8T cells), we assumed the “gold standard” cell composition to be 100% CD8T cells and 0% for the other cell types. We fit our model to *N*=44 purified cell types measured on the WGBS platform

We evaluated our platform-agnostic approach using WGBS reference methylome data from the BLUEPRINT Epigenome Database [23]. We downloaded *N*=44 samples from seven purified whole blood cell types, specifically B cells, CD4T cells, CD8T cells, neutrophils, eosinophils, monocytes, and natural killer cells. For a given WGBS sample (e.g., CD8T cells), we assumed the “gold standard” cell composition to be 100% CD8T cells and 0% for the other cell types. We fit our model to *N*=44 purified cell types measured on the WGBS platform. We found our platform-agnostic approach closely matches the expected cell composition estimates from the purified whole blood WGBS samples (Fig. 5).

Next, we used the BLUEPRINT reference methylomes to construct a set of cell type-specific DMRs to investigate whether DMRs identified with the purified cell types measured on the WGBS platform can lead to improved estimates of cell composition with methylCC as opposed to DMRs identified with the purified cell types measured on the Illumina 450K array platform. Using the WGBS “gold standard” data, we found the DMRs identified with the purified cell types measured on the WGBS platform resulted performed better than DMRs identified with the purified cell types measured on the Illumina 450K array platform (Additional file 1: Figure S3). Using the *two-platform dataset*, we found methylCC results in a substantial improvement over the Houseman approach with either set of DMRs, but using the DMRs identified with the 450K reference methylomes performs slightly better (Additional file 1: Figure S4). Data exploration of the BLUEPRINT data reveals that this is likely due to a batch effect in the BLUEPRINT data (Additional file 1: Figure S5).

### methylCC accurately estimates cell composition of DNAm samples measured on Illumina 450K array platforms

To validate the results of our simulation study using whole blood samples measured on the Illumina 450K array platform, we compared the cell composition estimates from our model and the Houseman model using two publicly available data sets with DNAm whole blood samples measured on the Illumina 450K array platform. In the first data set [24], the *N*=78 whole blood samples had their cell composition independently estimated using flow cytometry, which can be considered as a “gold standard” [22]. We found our platform-agnostic approach closely matches the independent cell composition measurements (Additional file 1: Figure S6).

Next, we used a second data set [25] with *N*=689 whole blood samples, which did not have independent measurements of cell composition. Here, we considered the cell composition estimates from the Houseman model to be the “gold standard” for the purposes of this assessment because the Houseman model was specifically designed for the Illumina 450K array platform and it has been previously considered as a “gold standard" [22]. Using this data, we found our platform-agnostic approach closely matches the referenced-based approach (Additional file 1: Figure S7).

## Discussion

A major challenge in measuring DNAm is variability introduced from intra-sample cellular heterogeneity, which is a convolution of DNAm profiles across cell types. This is particularly problematic in epigenome-wide association studies for human disease performed on whole blood, a heterogeneous tissue. Accounting for this source of variability is a first step to determine the actual cell proportions of each sample. Currently, the most effective approach is based on fitting a linear model in which one assumes the DNAm profiles of the representative cell types are known for a specific platform technology, the Illumina microarray platform. Although this method works well in practice, we have demonstrated that if the DNAm data was generated on a new platform technology, such as RRBS or WGBS, this can lead to technology-specific biases in the cell composition estimates.

To address this, we have developed a latent variable model with region-specific and platform-dependent random effects to accurately estimate the cell composition in DNAm whole blood samples measured from any platform technology. By using informative genomic regions that are either methylated or unmethylated for each purified cell type, our model can account for the platform-specific biases directly and model methylation states using latent variables. We have illustrated how we can estimate the cell composition across platform technologies as cell types preserve their methylation state in regions independent of platform, despite observed measurements being platform-dependent. Note that, our current model assumes that the random effects and measurement error are normally distributed. Although these assumptions were a practical approximation that led to an improvement for RRBS data and accurately identified purified cell types in WGBS data, the model may need to be generalized to other distributions, such as count data for which negative binomial models may be more appropriate. Given that sequencing platform technologies are poised to become more widely used for studies measuring DNAm in whole blood, this suggests that our method is an needed contribution.

## Conclusions

We demonstrated that our method accurately estimates the cell composition from whole blood samples and is applicable across multiple platforms, including microarray and sequencing platforms. Specifically, we illustrated how our method significantly improves the estimates of the cell composition compared to the reference-based method in whole blood samples measured on a sequencing platform using real and simulated whole blood samples, in addition to purified whole blood cell types measured on a sequencing platform. Our approach is agnostic to platform because it first uses experimental data to identify regions in which each cell type is clearly methylated or unmethylated, and then models these as latent states. While the continuous measurements used in the linear model approaches are affected by platform-specific biases, the latent states are biologically driven and therefore technology independent, implying that experimental data only needs to be collected once. We have implemented our method into the *methylCC* R-package providing researchers a tool to estimate the cell composition in the analysis of their own whole blood DNAm data.

## Methods

### Using cell sorted experimental data to identify informative genomic regions in ***Z***

Cell sorted experimental data is needed to identify *R* informative genomic regions for which the *k*th cell type is either clearly methylated (*Z*_*rk*_=1) or not methylated (*Z*_*rk*_=0) for regions *r*∈(1,…,*R*). This is step is done only once for each type of heterogeneous (biological) sample, such as whole blood, and does not depend on the platform technology. In addition, this matrix ***Z*** needs to be full rank for the parameters of interest, *π*_*ik*_ to be identifiable.

In application for estimating the cell composition in whole blood samples, we used cell sorted data described in [1], which were measured on the Illumina 450K array platform. This dataset includes six biological replicates for each of the six purified cell type (B cells, monocytes, granulocytes, CD8T cells, CD4T cells, and natural killer (NK) cells). We used the bumphunter [26] Bioconductor [27] package to identify differentially methylated regions (DMRs) across cell types. For example, to search for DMRs such that the six granulocytes samples are unmethylated and the other cell types are methylated (Fig. 3), we fit a linear model *Y*_*ij*_=*β*_0_(*l*_*j*_)+*β*_1_(*l*_*j*_)*X*_*j*_+*ε*_*ij*_ at each *j*th genomic position (or CpG site) where *Y*_*ij*_ represents observed DNAm level in the *i*^*th*^ biological replicate for a purified cell type at position *j* with a covariate of interest, *X*_*j*_, (for example *X*_*j*_=0 for granulocytes and *X*_*j*_=1 for other cell types). Then, we searched for regions of CpGs such that *β*_1_(*l*)≠0. For more details on identifying DMRs, we refer the reader to [26, 28].

We searched for regions that were not overlapping so they would be considered independent observations. In certain pairwise cell type comparisons, the only regions found contained just one CpG; however, we prioritized regions with more than one CpG whenever possible. In addition to these cell type-specific DMRs, our method has the option for a user to search for and include additional cell type-specific CpGs along with the DMRs, if too few DMRs are found. Following these steps, we identified *R*=210 regions satisfying our criteria (Additional file 1: Figure S1).

We also used the reference methylomes from the BLUEPRINT Epigenome Database (http://www.blueprint-epigenome.eu) [23], which contained *N*=44 samples from seven purified whole blood cell types, specifically B cells, CD4T cells, CD8T cells, neutrophils, eosinophils, monocytes, and natural killer cells. We combined neutrophils, eosinophils as one group called granulocytes. We used the bsseq [29] Bioconductor [27] package to store the WGBS data and we used the dmrseq [30] package to identify DMRs across the six cell types.

In the next section, we describe our estimation procedure to obtain the cell composition estimates, ***π***_*i*_=(*π*_*i*1_,…,*π*_*iK*_), and we note that we assume these regions ***Z*** are known here. This is because if we fit the model only to these regions, then the estimation procedure reduces to a missing data problem with random effects ***δ***_0_ and ***δ***_1_.

### Estimation procedure

Using the *R*=210 informative genomic regions identified above, we estimate the parameters of interest, namely the proportion of cell types ***π***_*i*_=(*π*_*i*1_,…,*π*_*iK*_) for the *i*∈(1,…,*N*) individuals, and the parameters $\boldsymbol {\theta } = \left (\alpha _{0}, \alpha _{1}, \sigma _{0}^{2}, \sigma _{1}^{2}, \tau ^{2}\right)$ in the proposed latent variable model (Eq. 2) using an EM algorithm with constraints $\sum _{k=1}^{K} \pi _{ik} = 1$ and *π*_*ik*_≥0 for all *k*.

#### Obtain initial parameter estimates ***θ***^(0)^ and $\boldsymbol {\pi }_{i}^{(0)}$ at step *t*=0

To obtain initial parameter estimates for the $\alpha _{0}^{(0)}$ and $\left (\sigma _{0}^{2}\right)^{(0)}$ at step *t*=0, we use the reference cell sorted dataset [1], which has six biological replicates for each cell type, to identify a set of *R*^0^ genomic regions that are clearly unmethylated (*Z*_*rk*_=0) in all *K* purified whole blood cell types. In these unmethylated regions, the expected DNAm level is

$$E(Y_{ir}) = \sum_{k=1}^{K} \pi_{ik} E(\delta_{0,r}) + E(\varepsilon_{ir}) = \sum_{k=1}^{K} \pi_{ik} \alpha_{0} = \alpha_{0} $$

and we use Jensen’s inequality to estimate an upper bound on the variance of *Y*_*ir*_:

$$\begin{aligned} Var(Y_{ir}) &= Var\left(\sum_{k=1}^{K} \pi_{ik} \delta_{0,r}\right) + Var(\varepsilon_{ir})\\ &\leq \sum_{k=1}^{K} \pi_{ik} Var(\delta_{0,r}) + Var(\varepsilon_{ir}) = \sigma_{0}^{2} + \tau^{2} \end{aligned} $$

Therefore, we obtain initial parameter estimates

$$\begin{aligned} \hat{\alpha}_{0}^{(0)} &= \frac{1}{N} \sum_{i=1}^{N} \left[ \frac{1}{R^{0}} \sum_{r=1}^{R^{0}} Y_{ir} \right] \\ (\hat{\sigma}_{0}^{2})^{(0)} &\geq \frac{1}{N} \sum_{i=1}^{N} \left[ \frac{1}{R^{0}-1} \sum_{r=1}^{R^{0}} (Y_{ir} -\bar{Y}_{i})^{2} \right] \end{aligned} $$

where the measurement error *τ*^2^ is assumed to be small. The argument is similar for the initial parameter estimates of $\alpha _{1}^{(0)}$ and $\left (\sigma _{1}^{2}\right)^{(0)}$ by identifying genomic regions (*R*^1^) where the CpGs are all methylated (*Z*_*rk*_=1) for all *K* purified cell types.

To obtain initial parameter estimates for the proportion of cell types, $\boldsymbol {\pi }_{i}^{(0)}$ with constraints $\sum _{k=1}^{K} \pi _{ik}^{(0)} = 1$ and $\pi _{ik}^{(0)} \geq 0$, we use the fact that $\hat{{\pi}}_{i} = \text{argmin}_{{\pi}_{i}} \log L = \text{argmax}_{{\pi}_{i}} (-\log L)$ and − log*L*∝(*Y*−*Xπ*)^*T*^(*Y*−*Xπ*). This non-negative least squares (NNLS) problem with constraints is equivalent to the quadratic programming problem $\text{argmin}_{{\pi}_{i}} \left (\frac {1}{2} {\pi}_{i}^{T} Q{\pi}_{i} + a^{T} {\pi}_{i}\right)$ where *Q*=(*X*^*T*^*X*) and *a*=(−*X*^*T*^*Y*) [31, 32]. Therefore, we calculate $\boldsymbol {\hat {X}}^{(0)} = (1-\boldsymbol {Z}) \hat {\alpha }_{0}^{(0)} + \boldsymbol {Z} \hat {\alpha }_{1}^{(0)}$ and apply quadratic programming [31, 32] to solve for $ \hat {\boldsymbol {\pi }}_{i}^{(0)}= \left (\pi _{i1}^{(0)}, \ldots, \pi _{iK}^{(0)}\right)$. We use the solve.QP() function from the R package quadprog [33] to implement the quadratic programming. Finally, to obtain an initial parameter estimate for (*τ*^2^)^(0)^, we calculate

$$(\hat{\tau}^{2})^{(0)} = \frac{1}{RN} \sum_{i=1}^{N} \sum_{r=1}^{R} \left(Y_{ir} - \sum_{k=1}^{K} \hat{X}_{rk}^{(0)} \hat{\pi}_{ik}^{(0)}\right)^{2} $$

#### EM algorithm to estimate ***θ*** and ***π***

To construct an EM algorithm to obtain maximum likelihood estimates of ***θ*** and ***π***, we define the complete-data vector ***Y***^∗^=(***Y***,***δ***_0_,***δ***_1_) where ***Y***=(***Y***_1_,…,***Y***_*N*_) represents the observed DNAm levels for individuals *i*∈(1,…,*N*) each of length *R* regions. The complete-data likelihood is given by

$$f(\boldsymbol{Y}^{*} | \boldsymbol{\theta}, \boldsymbol{\pi}) = \prod_{i=1}^{N} f_{1}(\boldsymbol{Y}_{i} | \boldsymbol{\delta}_{0}, \boldsymbol{\delta}_{1}, \boldsymbol{\theta}, \boldsymbol{\pi}_{i}) f_{2}(\boldsymbol{\delta}_{0} | \boldsymbol{\theta}) f_{3}(\boldsymbol{\delta}_{1} | \boldsymbol{\theta}) $$

where $f_{1} \sim N\left (\sum _{k=1}^{K} \pi _{ik} \{(1-\boldsymbol {Z}_{k}) \boldsymbol {\delta }_{0} + \boldsymbol {Z}_{k} \boldsymbol {\delta }_{1}\}, \tau ^{2} I_{(R \times R)}\right), f_{2} \sim N\left (\alpha _{0}, \sigma _{0}^{2} I_{(R \times R)}\right)$, and $f_{3} \sim N\left (\alpha _{1}, \sigma _{1}^{2} I_{(R \times R)}\right)$. It is easy to show the log of the complete-data likelihood is linear in the following complete-data sufficient statistics: $T_{1} = \sum _{r=1}^{R} \delta _{0,r}, T_{2} = \sum _{r=1}^{R} \delta _{1,r}, T_{3} = \sum _{r=1}^{R} (\delta _{0,r})^{2}, T_{4} = \sum _{r=1}^{R} (\delta _{1,r})^{2}$, and $T_{5} = \sum _{r=1}^{R} (u_{ir})^{2}$ where $u_{ir} = Y_{ir} - \sum _{k=1}^{K} \pi _{ik} \{(1-Z_{rk}) \delta _{0,r} + Z_{rk} \delta _{1,r}\}$.

The EM algorithm alternates between the following two steps:

1. **E-Step**
  We can consider the two joint distributions ***Y***^∗^=(***Y***,***δ***_0_) and ***Y***^∗^=(***Y***,***δ***_1_) separately since ***δ***_0_ and ***δ***_1_ are independent. The joint distributions are also normally distributed
  $${}{\begin{aligned} \boldsymbol{Y}^{*} = (\boldsymbol{Y}, \boldsymbol{\delta}_{0}) \sim N \left(\left[ \begin{array}{c} \boldsymbol{X} \boldsymbol{\pi} \\ \alpha_{0} \boldsymbol{1} \end{array} \right]_{((RN + R) \times 1)}, \left[ \begin{array}{cc} \Sigma_{11} & \Sigma_{12} \\ \Sigma_{21} & \Sigma_{22} \\ \end{array} \right]_{((RN+R) \times (RN+R))} \right) \end{aligned}}$$
  $${}{\begin{aligned} \boldsymbol{Y}^{*} = (\boldsymbol{Y}, \boldsymbol{\delta}_{1}) \sim N \left(\left[ \begin{array}{c} \boldsymbol{X} \boldsymbol{\pi} \\ \alpha_{1} \boldsymbol{1} \end{array} \right]_{((RN + R) \times 1)}, \left[ \begin{array}{cc} \Sigma_{11} & \Sigma_{12} \\ \Sigma_{21} & \Sigma_{22} \\ \end{array} \right]_{((RN+R) \times (RN+R))} \right) \end{aligned}}$$
  where ***Y*** is a matrix of dimension *R*×*N*, but we convert this into a vector of length *RN*, ***X***=(1−***Z***)*α*_0_+***Z****α*_1_ is an *R*×*K* matrix and ***π*** is a *K*×*N* matrix. We convert the ***Xπ*** matrix into a vector of length *RN*. To derive the conditional distributions of ***δ***_0_|***Y*** and ***δ***_1_|***Y***, we use Theorem 3.2.3 and 3.2.4 in [34]:
  $${}{\begin{aligned} \boldsymbol{\delta}_{0} | \boldsymbol{Y} \sim N\left(\alpha_{0} \boldsymbol{1} + \Sigma_{21} \Sigma_{11}^{-1} [\boldsymbol{Y} - \boldsymbol{X} \boldsymbol{\pi}], \Sigma_{22} - \Sigma_{21} \Sigma_{11}^{-1} \Sigma_{12}\right) \end{aligned}} $$
  where
  ***X***=(1−***Z***)*α*_0_+***Z****α*_1_ is an *R*×*K* matrix. ***π*** is a *K*×*N* matrix.
  *Σ*_11_=*Cov*(***Y***) is an *RN*×*RN* covariance matrix with entries
    $${}{\begin{aligned} Cov(Y_{ir}, Y_{i^{'}r^{'}}) & = W_{0ri}^{2} \sigma_{0}^{2} + W_{1ri}^{2} \sigma_{1}^{2} + \tau^{2} & \texttt{if}\ r=r^{'}, i=i^{'} \\ & = W_{0ri} W_{0ri^{'}} \sigma_{0}^{2} + W_{1ri} W_{1ri^{'}} \sigma_{1}^{2} &\texttt{if}\ r=r^{'}, i \neq i^{'} \\ & = 0 & \texttt{if}\ r\neq r^{'}, i \neq i^{'} \end{aligned}} $$
    where $W_{0ri} = \sum _{k=1}^{K} \pi _{ik} (1-Z_{rk})$, and $W_{1ri} = \sum _{k=1}^{K} \pi _{ik} Z_{rk}$
  *Σ*_12_=*Cov*(***Y***,***δ***_0_) is an *RN*×*R* covariance matrix with entries
    $$\begin{array}{@{}rcl@{}} Cov(Y_{ir}, \delta_{0,r^{'}}) & = & W_{0ri} \sigma_{0}^{2} \hspace{.5in} \texttt{if}\ r=r^{'} \\ & = & 0 \hspace{0.87in} \texttt{if}\ r\neq r^{'} \end{array} $$
    Note: $\Sigma _{12}^{T} = \Sigma _{21}$.
  *Σ*_22_=*Cov*(***δ***_0_) is an *R*×*R* matrix with $Var(\delta _{0,r}) = \sigma _{0}^{2}$ and $\phantom {\dot {i}\!}Cov(\delta _{0,r}, \delta _{0,r^{'}}) = 0$
  We use the conditional distribution ***δ***_0_|***Y*** to calculate the *t*th iteration in the E-Step when computing *E*_*θ*_(*T*_1_|***Y***) and *E*_*θ*_(*T*_3_|***Y***).
  $${}{\begin{aligned}T_{1}^{(t)} & = \sum_{r=1}^{R} \left[ \hat{\alpha}_{0}^{(t)} \boldsymbol{1} + \hat{\Sigma}_{21}^{(t)} \left(\hat{\Sigma}_{11}^{(t)}\right)^{-1} \left[\boldsymbol{Y}_{i} - \left\{(1-\boldsymbol{Z}) \hat{\alpha}_{0}^{(t)} + \boldsymbol{Z} \hat{\alpha}_{1}^{(t)} \right\} \hat{\boldsymbol{\pi}}\right] \right] \\ T_{3}^{(t)} & = \sum_{r=1}^{R} \left[ \hat{\alpha}_{0}^{(t)} \boldsymbol{1} + \hat{\Sigma}_{21}^{(t)} \left(\hat{\Sigma}_{11}^{(t)}\right)^{-1} [\boldsymbol{Y}_{i} - \left\{(1-\boldsymbol{Z}) \hat{\alpha}_{0}^{(t)} + \boldsymbol{Z} \hat{\alpha}_{1}^{(t)} \right\} \hat{\boldsymbol{\pi}}] \right]^{2}\\ &\quad+ diag\left(\hat{\Sigma}_{22}^{(t)} - \hat{\Sigma}_{21}^{(t)} \left(\hat{\Sigma}_{11}^{(t)}\right)^{-1} \hat{\Sigma}_{12}^{(t)} \right) \end{aligned}} $$
  Similarly, we can show
  $${}\boldsymbol{\delta}_{1} | \boldsymbol{Y} \sim N\left(\alpha_{1} \boldsymbol{1} + \Sigma_{21} \Sigma_{11}^{-1} [\boldsymbol{Y} - \boldsymbol{X} \boldsymbol{\pi}], \Sigma_{22} - \Sigma_{21} \Sigma_{11}^{-1} \Sigma_{12}\right) $$
  where
  ***X***=(1−***Z***)*α*_0_+***Z****α*_1_ is an *R*×*K* matrix. ***π*** is a *K*×*N* matrix.
  *Σ*_11_=*Cov*(***Y***) is same as defined above.
  *Σ*_12_=*Cov*(***Y***,***δ***_1_) is an *RN*×*R* covariance matrix with entries
    $$\begin{array}{@{}rcl@{}} Cov(Y_{ir}, \delta_{1,r^{'}}) & = & W_{1ri} \sigma_{1}^{2} \hspace{.5in} \texttt{if}\ r=r^{'} \\ & = & 0 \hspace{0.87in} \texttt{if}\ r\neq r^{'} \end{array} $$
    Note: $\Sigma _{12}^{T} = \Sigma _{21}$.
  *Σ*_22_=*Cov*(***δ***_1_) is an *R*×*R* matrix with $Var(\delta _{1,r}) = \sigma _{1}^{2}$ and $\phantom {\dot {i}\!}Cov(\delta _{1,r}, \delta _{1,r^{'}}) = 0$
  We use the conditional distribution ***δ***_1_|***Y*** to calculate the *t*^*th*^ iteration in the E-Step when computing *E*_*θ*_(*T*_2_|***Y***) and *E*_*θ*_(*T*_4_|***Y***).
  $${}{\begin{aligned} T_{2}^{(t)} & = \sum_{r=1}^{R} \left[ \hat{\alpha}_{1}^{(t)} \boldsymbol{1} + \hat{\Sigma}_{21}^{(t)} \left(\hat{\Sigma}_{11}^{(t)}\right)^{-1} \left[\boldsymbol{Y} - \left\{(1-\boldsymbol{Z}) \hat{\alpha}_{0}^{(t)} + \boldsymbol{Z} \hat{\alpha}_{1}^{(t)} \right\} \hat{\boldsymbol{\pi}}\right] \right] \\ T_{4}^{(t)} & = \sum_{r=1}^{R} \left[ \hat{\alpha}_{1}^{(t)} \boldsymbol{1} + \hat{\Sigma}_{21}^{(t)} \left(\hat{\Sigma}_{11}^{(t)}\right)^{-1} \left[\boldsymbol{Y} - \left\{(1-\boldsymbol{Z}) \hat{\alpha}_{0}^{(t)} + \boldsymbol{Z} \hat{\alpha}_{1}^{(t)} \right\} \hat{\boldsymbol{\pi}}\right] \right]^{2}\\&\quad + diag\left(\hat{\Sigma}_{22}^{(t)} - \hat{\Sigma}_{21}^{(t)} \left(\hat{\Sigma}_{11}^{(t)}\right)^{-1} \hat{\Sigma}_{12}^{(t)} \right) \end{aligned}} $$
2. **M-Step**
  The complete-data maximum likelihood estimates (MLEs) were calculated by using the log of the complete-data likelihood, taking the derivative with respect to the individual parameters, setting the likelihood equal to zero and solving for the MLEs.
  $$\begin{array}{@{}rcl@{}} \hat{\alpha}_{0} & = & \frac{T_{1}}{R} \\ \hat{\alpha}_{1} & = & \frac{T_{2}}{R} \\ \hat{\sigma}_{0}^{2} & = & \frac{T_{3}}{R} - (\hat{\alpha}_{0})^{2} \\ \hat{\sigma}_{1}^{2} & = & \frac{T_{4}}{R} - (\hat{\alpha}_{1})^{2} \end{array} $$
  Using these MLEs, we can substitute the sufficient statistics calculated in the E-Step:
  $$\begin{array}{@{}rcl@{}} \hat{\alpha}_{0}^{(t+1)} & = & \frac{T_{1}^{(t)}}{R} \\ \hat{\alpha}_{1}^{(t+1)} & = & \frac{T_{2}^{(t)}}{R} \\ (\hat{\sigma}_{0}^{2})^{(t+1)} & = & \frac{T_{3}^{(t)}}{R} - \left(\hat{\alpha}_{0}^{(t+1)}\right)^{2} \\ (\hat{\sigma}_{1}^{2})^{(t+1)} & = & \frac{T_{4}^{(t)}}{R} - \left(\hat{\alpha}_{1}^{(t+1)}\right)^{2} \end{array} $$
  To estimate ***π***_*i*_, we apply quadratic programming [31, 32] (see section on “[Sec Sec15]” for details) with the constraints $\sum _{k=1}^{K} \pi _{ik} = 1$ and *π*_*ik*_≥0 for all *k*. We calculate ***X***^(*t*)^ using the *t*^*th*^ iteration of the conditional expectations *E*_*θ*_(***δ***_0_|***Y***) and *E*_*θ*_(***δ***_1_|***Y***) then apply quadratic programming [31, 32] to solve for $ \hat {\boldsymbol {\pi }}_{i}^{(t+1)}= \left (\pi _{i1}^{(t+1)}, \ldots, \pi _{iK}^{(t+1)}\right)$. We use the solve.QP() function from the R package quadprog [33] to implement the quadratic programming.
  Finally, the MLE for *τ*^2^, was calculated by using the log of the complete-data likelihood, taking derivative with respect to *τ*^2^, setting likelihood equal to zero and solving.
  $$(\hat{\tau}^{2})^{(t+1)} = \frac{1}{R*N} \sum_{i=1}^{N} \sum_{r=1}^{R} \left(Y_{ir} - \sum_{k=1}^{K} X_{rk}^{(t)} \pi_{ik}^{(t+1)}\right)^{2} $$

### Details for simulation studies

We created platform-dependent cell type-specific DNAm profiles for the *k*^*th*^ cell type ($\boldsymbol {X}_{k}^{450K}$ and $\boldsymbol {X}_{k}^{RRBS}$) where $\boldsymbol {X}_{k}^{*} = (1-\boldsymbol {Z}_{k}) \boldsymbol {\delta }_{0}^{*} + \boldsymbol {Z}_{k} \boldsymbol {\delta }_{1}^{*}$ by simulating platform-dependent random effects $\left (\boldsymbol {\delta }_{l}^{*} \sim N(\alpha _{l}^{*}, (\sigma _{l}^{2})^{*} I_{(R \times R)}\right)$ for both *l*=0,1 (Fig. 2b). For each whole blood DNAm sample (*N*=200), we simulate a relative proportion of cell types (***π***_*i*_) and measurement error (***ε***_*i*_) to create the observed DNAm level in the 450k array platform $\left (\boldsymbol {Y}_{i}^{450k} = \sum _{k=1}^{K} \boldsymbol {\pi }_{ik} \boldsymbol {X}_{k}^{450k} + \boldsymbol {\varepsilon }_{i}\right)$ and the RRBS platform $\left (\boldsymbol {Y}_{i}^{RRBS} = \sum _{k=1}^{K} \boldsymbol {\pi }_{ik} \boldsymbol {X}_{k}^{RRBS} + \boldsymbol {\varepsilon }_{i}\right)$.

### Assessment of performance

Next, we estimate the cell composition of, for example, the 450k array and RRBS samples using both the reference-based Houseman method and our platform-agnostic method. We do not scale the cell compositions estimates to 1 to allow for potential unaccounted cell types.

We calculate the cell type-specific *RMSE*_*k*_ as

$$RMSE_{k} = \sqrt{\frac{1}{N} \sum_{i=1}^{N} (\hat{\pi}_{ik} - \pi_{ik})^{2}} $$

where *π*_*ik*_ is the true cell composition and $\hat {\pi }_{ik}$ is the estimated cell composition (using either Houseman model or our proposed model) in the *i*^*th*^ sample and *k*th cell type. The cell type-specific *RMSE*_*k*_ is averaged across cell types and recorded as the mean RMSE. We repeat the above *n*_*sims*_=100 times to calculate the distribution of mean RMSE.

## Supplementary information

**Additional file 1** Supplementary Figures S1-S7.
