# Supplementary material for: methylCC: technology-independent estimation of cell type composition using differentially methylated regions
Source: Genome Biol. 2019 Nov 29;20:261. doi: 10.1186/s13059-019-1827-8 (PMC6883691; doi:10.1186/s13059-019-1827-8)
Supplement: Supplementary file 1 — Additional file 1 Supplementary Figures S1-S7. [file 13059_2019_1827_MOESM1_ESM.pdf]

Additional file 1

Supplementary Figures

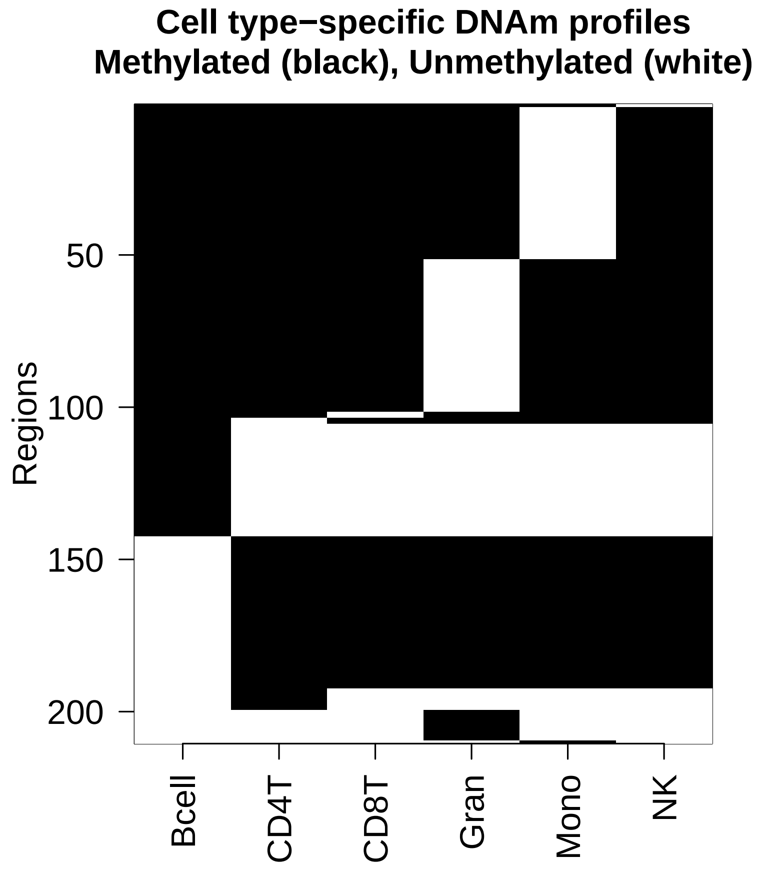

Figure S1 Cell type-specific DNAm profiles in whole blood. The image contains the informative genomic regions (R=210) containing regions that are either methylated (black) or unmethylated (white) in B-cells, CD4T-cells, CD8T-cells, Granulocytes (Gran), Monocytes (Mono) and Natural Killer (NK) cells.

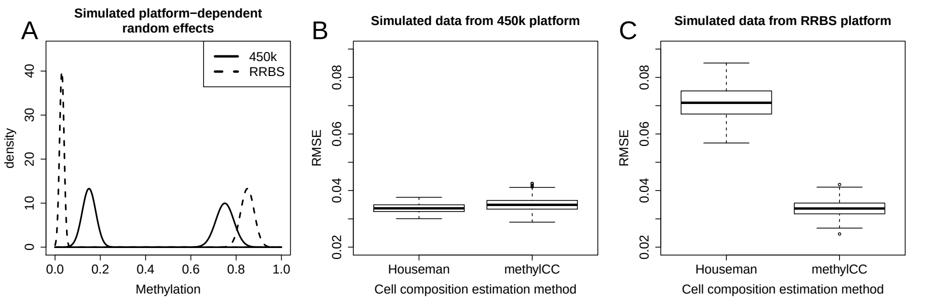

Figure S2 Simulation study illustrating improvement in RMSE by accounting for platform-dependent effects. (A) Simulated platform-dependent random effects, similar to the platform-dependent effects observed in Illumina 450K and RRBS data (Figure ??B). Root mean squared error (RMSE) averaged across cell types for whole blood DNAm samples measured on (B) the Illumina 450k array platform and on (C) the RRBS platform. Within each plot, the RMSE is shown for the reference-based method from Houseman et al. (2012) [1] and our proposed model (methylCC).

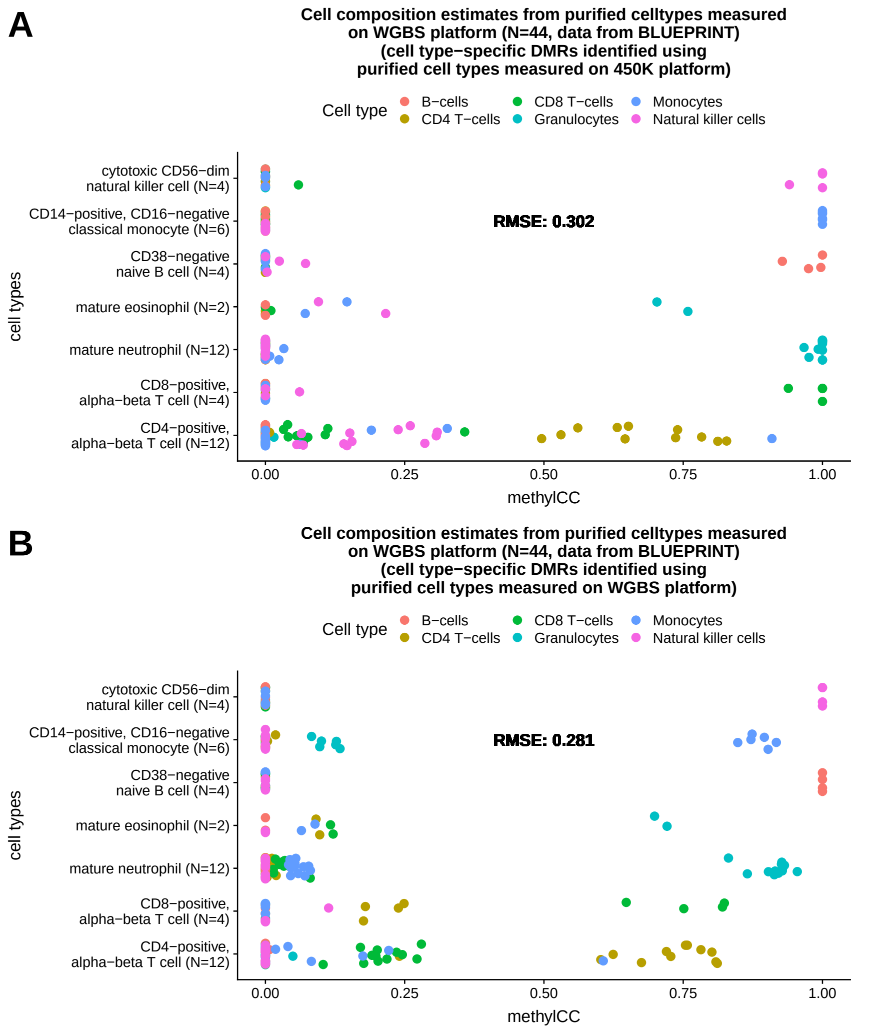

Figure S3 methylCC is agnostic to the platform technology used to identify differentially methylated regions in the reference methylomes. The top figure is identical to Figure 5 and the bottom plot is new, but we reproduce both here for comparison. These are cell composition estimates from N=44 WGBS samples from the BLUEPRINT Epigenome Database [2] from seven purified whole blood cell types, specifically B-cells, CD4T-cells, CD8T-cells, neutrophils, eosinophils, monocytes, and natural killer cells. For a given WGBS sample (e.g. CD8T-cells), we assumed the "gold standard" cell composition to be 100% CD8T-cells and 0% for the other cell types. We fit our model to $N = 44$ purified cell types measured on the WGBS platform. (A) methylCC using the cell type-specific DMRs identified using reference methylomes measured on the Illumina 450K array platform (data from FlowSorted.Blood.450K [3] Bioconductor [4] package). (B) methylCC using the cell type-specific DMRs identified using reference methylomes measured on the WGBS platform (data from BLUEPRINT [2]).

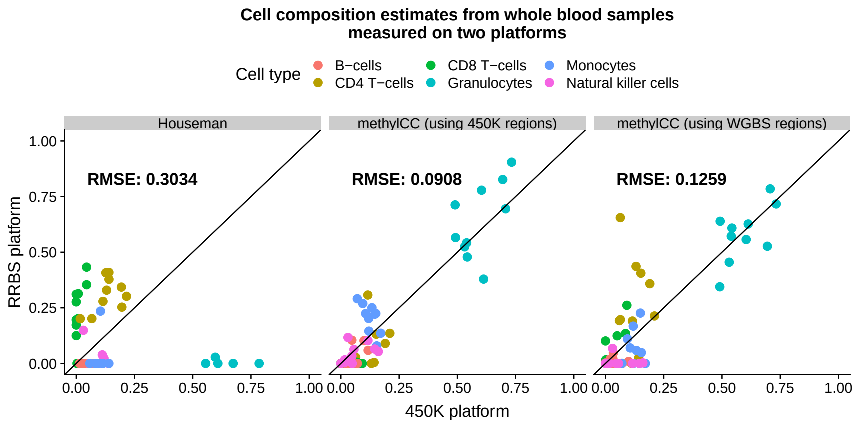

Figure S4 methylCC is agnostic to the platform technology used to identify differentially methylated regions in the reference methylomes. The left and middle figure are identical to Figure 4 and the right figure is new, but we reproduce all three here for comparison. These are cell composition estimates from N=10 whole blood samples (K=6 cell types) measured on the Illumina 450k microarray platform (x-axis) and the RRBS platform (y-axis). Three methods were used to estimate the cell composition: (1) the model proposed by Houseman et al. (2012) [1], as implemented by Jaffe et al. (2014) [5], that was developed for samples measured on the Illumina 450k microarray platform (left), (2) methylCC using DMRs identified using the reference methylomes measured on the Illumina 450K array platform (data from FlowSorted.Blood.450K [3] Bioconductor [4] package) (middle), and (3) methylCC using DMRs identified using the reference methylomes measured on the WGBS platform (data from BLUEPRINT [2]) (right). Root mean squared error (RMSE) is the difference between the cell composition estimates for whole blood DNAm samples measured on the Illumina 450k array platform and on the RRBS platform (averaged across cell types). Within each plot, the RMSE is shown for the reference-based method from Houseman et al. (2012) [1] (left) and our proposed model (methylCC) (middle and right) using two sets of reference methylomes measured on 450K and WGBS platforms, respectively.

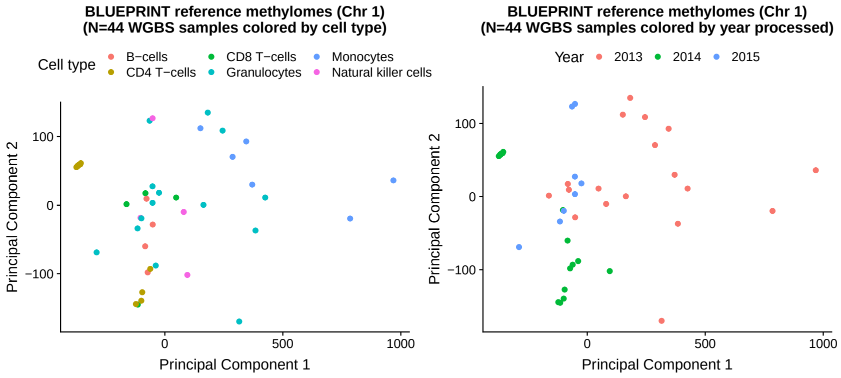

Figure S5 Principal Components Analysis with BLUEPRINT reference methylomes. Using CpGs in Chromosome 1, we applied Principal Components Analysis (PCA) and compared how the N=44 purified cell types measured on the WGBS platform from BLUEPRINT [2] cluster along the first two principal components (PCs) colored by (A) the purified cell type samples and (B) the year the samples were processed.

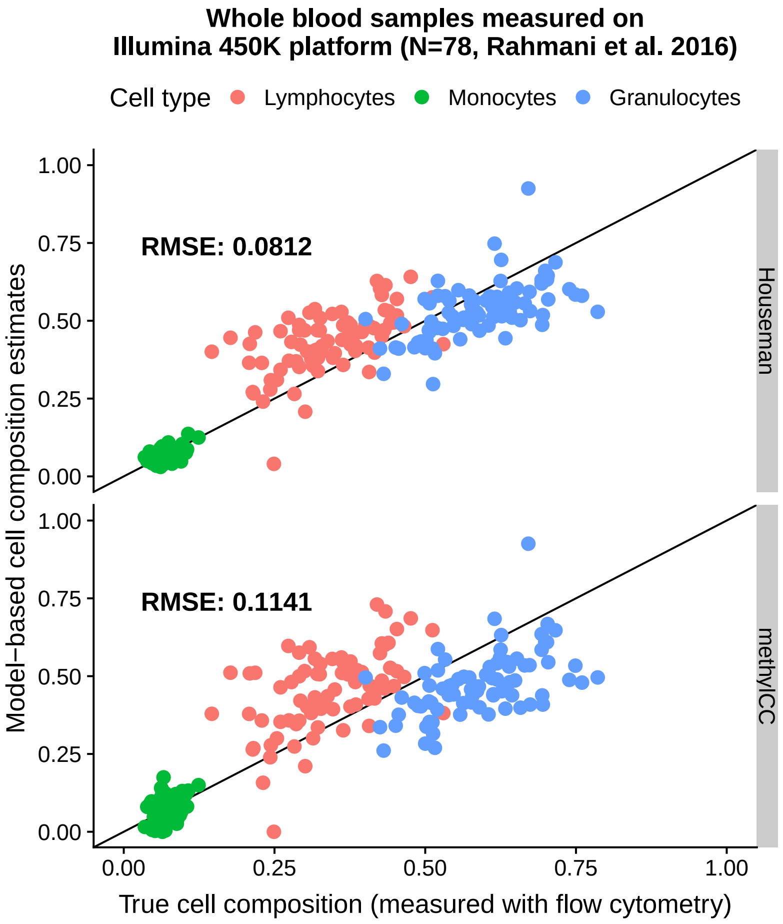

Figure S6 Platform-agnostic approach and reference-based approach closely matches the true cell composition using data measured on the Illumina 450K platform. Cell composition estimates (K=6 cell types) for N=78 whole blood samples from Rahmani et al. (2016) [6] from the Gala II population [7] measured on the Illumina 450K array platform. Here, the true cell composition for each whole blood sample was independently measured using flow cytometry and is considered the “gold standard”. We compared the true cell composition to cell composition estimates (x-axis) from the reference-based approach (Houseman) (top y-axis) to our platform-agnostic approach (methylCC) (bottom y-axis). The Houseman and methylCC approaches return estimates for monocytes, granulocytes, Bcells, CD8T, CD4T and natural killer. However, we combined the last four cell types into lymphocytes because the cell composition measured using flow cytometry grouped cells into granulocytes, monocytes and lymphocytes. Root mean squared error (RMSE) is the difference between the true cell composition estimates (x-axis) and the model-based estimates (y-axis) for whole blood DNAm samples measured on the Illumina 450k array platform (averaged across cell types).

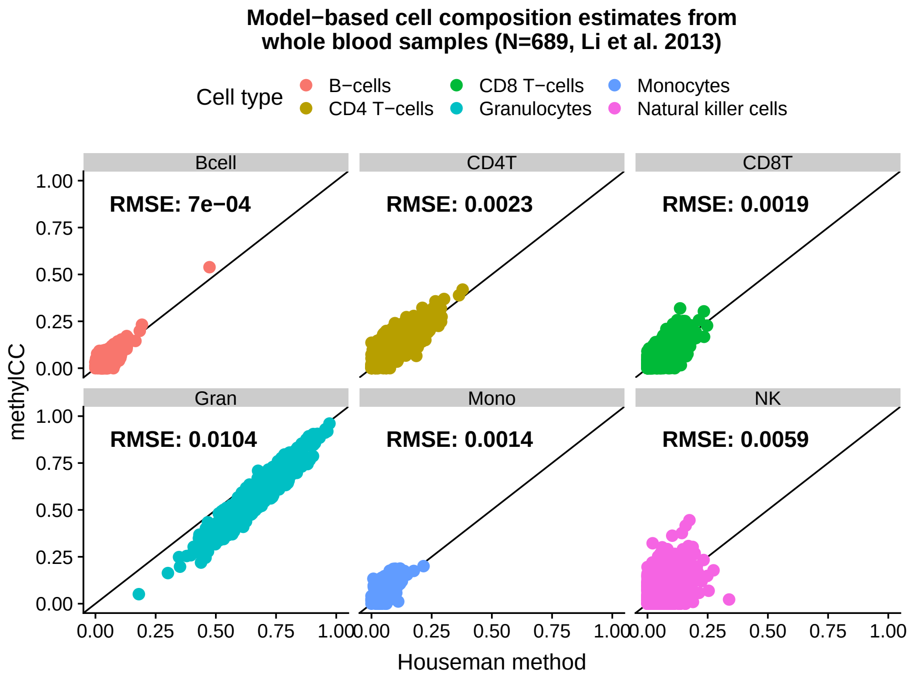

Figure S7 Platform-agnostic approach closely matches the reference-based approach using data measured on the Illumina 450K platform. Cell composition estimates (K=4 cell types) for N=689 whole blood samples from Liu et al. (2013) [8] measured on the Illumina 450K array platform. Here, the cell composition estimates from the Houseman method (x-axis) are considered the "gold standard" because this method was specifically designed for the Illumina 450K array platform. We compared this reference-based approach to cell composition estimates from our platform-agnostic approach (methylCC) (y-axis).

Author details

References

1. Houseman, E.A., Accomando, W.P., Koestler, D.C., Christensen, B.C., Marsit, C.J., Nelson, H.H., Wiencke, J.K., Kelsey, K.T.: DNA methylation arrays as surrogate measures of cell mixture distribution. BMC Bioinformatics **13**, 86 (2012). doi:10.1186/1471-2105-13-86
2. BLUEPRINT consortium: Quantitative comparison of DNA methylation assays for biomarker development and clinical applications. Nat Biotechnol **34**(7), 726–37 (2016). doi:10.1038/nbt.3605
3. Jaffe, A.E.: FlowSorted.Blood.450k: Illumina HumanMethylation Data on Sorted Blood Cell Populations. (2018). doi:10.18129/B9.bioc.FlowSorted.Blood.450k. R package version 1.20.0. https://www.bioconductor.org/packages/FlowSorted.Blood.450k
4. Huber, W., Carey, V.J., Gentleman, R., Anders, S., Carlson, M., Carvalho, B.S., Bravo, H.C., Davis, S., Gatto, L., Girke, T., Gottardo, R., Hahne, F., Hansen, K.D., Irizarry, R.A., Lawrence, M., Love, M.I., MacDonald, J., Obenchain, V., Oleś, A.K., Pagès, H., Reyes, A., Shannon, P., Smyth, G.K., Tenenbaum, D., Waldron, L., Morgan, M.: Orchestrating high-throughput genomic analysis with Bioconductor. Nat Methods **12**(2), 115–21 (2015). doi:10.1038/nmeth.3252
5. Jaffe, A.E., Irizarry, R.A.: Accounting for cellular heterogeneity is critical in epigenome-wide association studies. Genome Biol **15**(2), 31 (2014). doi:10.1186/gb-2014-15-2-r31
6. Rahmani, E., Zaitlen, N., Baran, Y., Eng, C., Hu, D., Galanter, J., Oh, S., Burchard, E.G., Eskin, E., Zou, J., Halperin, E.: Sparse PCA corrects for cell type heterogeneity in epigenome-wide association studies. Nat Methods **13**(5), 443–5 (2016). doi:10.1038/nmeth.3809
7. Pino-Yanes, M., Thakur, N., Gignoux, C.R., Galanter, J.M., Roth, L.A., Eng, C., Nishimura, K.K., Oh, S.S., Vora, H., Huntsman, S., Nguyen, E.A., Hu, D., Drake, K.A., Conti, D.V., Moreno-Estrada, A., Sandoval, K., Winkler, C.A., Borrell, L.N., Lurmann, F., Islam, T.S., Davis, A., Farber, H.J., Meade, K., Avila, P.C., Serebrisky, D., Bibbins-Domingo, K., Lenoir, M.A., Ford, J.G., Brigino-Buenaventura, E., Rodriguez-Cintron, W., Thyne, S.M., Sen, S., Rodriguez-Santana, J.R., Bustamante, C.D., Williams, L.K., Gilliland, F.D., Gauderman, W.J., Kumar, R., Torgerson, D.G., Burchard, E.G.: Genetic ancestry influences asthma susceptibility and lung function among Latinos. J Allergy Clin Immunol **135**(1), 228–35 (2015). doi:10.1016/j.jaci.2014.07.053
8. Liu, Y., Aryee, M.J., Padyukov, L., Fallin, M.D., Hesselberg, E., Runarsson, A., Reinius, L., Acevedo, N., Taub, M., Ronninger, M., Shchetynsky, K., Scheynius, A., Kere, J., Alfredsson, L., Klareskog, L., Ekström, T.J., Feinberg, A.P.: Epigenome-wide association data implicate DNA methylation as an intermediary of genetic risk in rheumatoid arthritis. Nat Biotechnol **31**(2), 142–7 (2013). doi:10.1038/nbt.2487
